# Advanced numerical investigation of the heat flux in an array of microbolometers

**DOI:** 10.1038/s41598-019-47472-2

**Published:** 2019-07-31

**Authors:** Matteo Stocchi, Davide Mencarelli, Luca Pierantoni, Alexander Göritz, Canan Baristiran Kaynak, Matthias Wietstruck, Mehmet Kaynak

**Affiliations:** 1 IHP Microelectronics, Im Technologiepark 25, 15236 Frankfurt (Oder), Germany; 2 Universitá Politecnica delle Marche, Ancona, 60131 Italy; 3 Istituto Nazionale di Fisica Nucleare (INFN) - Laboratori Nazionali di Frascati (LNF), Via E. Fermi, Frascati, Roma, Italy

**Keywords:** Electrical and electronic engineering, Information theory and computation

## Abstract

The investigation of the thermal properties of an array of microbolometers has been carried out by mean of two independent numerical analysis, respectively the Direct-Simulation Monte Carlo (DSMC) and the classic diffusive approach of the Fourier’s equation. In particular, the thermal dissipation of a hot membrane placed in a low-pressure cavity has been studied for different values of the temperature of the hot body and for different values of the pressure of the environment. The results for the heat flux derived from the two approaches have then been compared and discussed.

## Introduction

Due to the high level of rarefaction, the simulation of gas flow in a micro-cavity that encloses an array of microbolometers^1–5^ is much more challenging than in the classical flow regimes: the low number of gas molecules encapsulated in the considered domain make their mean free path much longer than usual, so the diffusive description used in the continuum approach (Navier-Stokes equations) ceases to be valid. The degree of rarefaction of a gas in such micro-systems is generally defined by the Knudsen parameter

1 $${K}_{n}=\frac{\lambda }{L},$$

where *λ* is the mean free path of the molecule and L the characteristic dimension of the system. Depending on the value of the Knudsen parameter, three different approaches may be used to characterize the phenomenon under investigation: for ${K}_{n}\le {10}^{-3}$ the classic diffusive approach gives the best results as the gas flow can still be assumed as a continuum, for ${10}^{-3}\le {K}_{n}\le {10}^{-1}$ the slip/transition flow (an extension of the diffusive approach for low pressure regime) should be used and, for ${K}_{n}\ge {10}^{-1}$, a discrete approach is needed since the system enters in the free molecular flow regime. Depending on the ratio between the collision probability between two different molecules and the collision probability between a molecule and a wall of the considered domain, the free molecular regime can further be sub-classified in the collision (${10}^{-1}\le {K}_{n}\le 10$) and collisionless (${K}_{n}\ge 10$) cases. The exact solution for systems characterized by high Knudsen number values is given by the Boltzmann equation, but for most of the real cases study this approach results too expensive in computational terms, so another way of addressing the problem is needed. The Direct-Simulation Monte Carlo (DSMC)^6–11^ is one of the most used and successful tracking simulation method for rarefied gas flow. The basic principle behind such approach is that each simulated body represents *n* real particles, so the overall solution is given by mean of statistical considerations. Although the DSMC provides a rigorous solution for finding the behaviour of the gas flow in a micro-cavity, it would be convenient to rely on a rather simpler and less time-consuming approach for addressing the optimization process of the system geometric parameters. An approximate solution is given by the rarefied gas heat transfer theory^12,13^, that makes use of the diffusive continuum approach by correcting the expression for the thermal conductivity evaluated at ambient pressure *k*_*amb*_ to consider the reduced pressure of the environment^14–16^. In what follows, a detailed report about the researches that have been made for evaluating the heat flux of a hot body in a low-pressure cavity will be presented, and a comparison between the two approaches of DSMC and Fourier’s equation will be given.

## Theoretical Background

According to the nature of the system under analysis, the DSMC method have been used for getting a reliable solution about the kinetic behaviour of the gas particle. The millimetric scale of the cavity that encloses the array of microbolometers and its low-pressure environment (*p*~10^−2^ mbar) lead to a Knudsen parameter well above the limit for the collisionless case, so any particle-particle interaction will be considered. For what concerns the particle-wall interaction, the fully diffusive approach with complete thermal accommodation has been chosen. According to the surface roughness of the cavity walls, the incident molecules can either suffer of multiple scattering, being momentarily trapped or even being fully absorbed. In such a case, the velocities of the particles that have been reflected by the cavity walls are completely uncorrelated to the velocities of the incident ones, and they must satisfy the Maxwellian velocity probability distribution for the particle valocity

2 $$f(c^{\prime} )={(\frac{m}{2\pi {k}_{B}{T}_{W}})}^{3/2}{e}^{-(\frac{m{c^{\prime} }^{,2}}{2{k}_{B}{T}_{W}})},$$

where *m* is the particle mass, *T*_*W*_ the wall temperature and *k*_*B*_ the Boltzmann’s constant. The reflected velocities ${u^{\prime} }_{n}$, ${u^{\prime} }_{t,1}$ and ${u^{\prime} }_{t,2}$ can then be sampled from (2) depending on the reflection plane orientation. In general:

3 $${u^{\prime} }_{n}=\pm \,{R}_{1}\sqrt{\frac{2{k}_{B}{T}_{W}}{m}},$$

4 $${u^{\prime} }_{t,1}={R}_{2}\,\cos (\theta )\sqrt{\frac{2{k}_{B}{T}_{W}}{m}},$$

5 $${u^{\prime} }_{t,2}={R}_{2}\,\sin (\theta )\sqrt{\frac{2{k}_{B}{T}_{W}}{m}},$$

where ${R}_{i}\sqrt{-\mathrm{ln}(r)}$ and *θ* = 2*πr*, with *r* being a uniform random number between 0 and 1. The definition of the heat flux comes from one of the basic principles of the molecular dynamics: by considering a generic wall element *i*, the heat flux *q*_*i*_ can be regarded as the ratio of the difference between the energy $\varepsilon$ of all the incoming and reflected particles *N* to the time *t* and the area of the considered surface *A*

6 $${q}_{i}=\frac{{\sum }_{j=1}^{N}\,{\varepsilon }_{j}^{I}-{\sum }_{j=1}^{N}\,{\varepsilon }_{j}^{R}}{tA}.$$

For what concerns the total energy associated to each particle, for the case of a monoatomic molecule the only contribution comes from the kinetic energy, while for the diatomic and polyatomic cases also the rotational energy must be considered.

The approximate approach, e.g. the one passing through the Fourier’s equation, makes use of a corrected expression for the conductivity *k*. If to consider the thermal conductivity of a generic material at ambient pressure *k*_*amb*_, its corresponding low-pressure form *k*′ is given as

7 $$k^{\prime} =\frac{{k}_{amb}}{1+\frac{\sqrt{2}\beta {k}_{B}T}{\pi {d}_{m}^{2}p{L}_{i}}},$$

where *d*_*m*_ is the diameter of the particle, *p* the pressure of the system, *L*_*i*_ the characteristic dimension of the considered domain and *β* is a constant between 1.5 and 2, which depends on the gas type, core material characteristics and mean temperature.

## Results and Discussion

**Figure 1:**
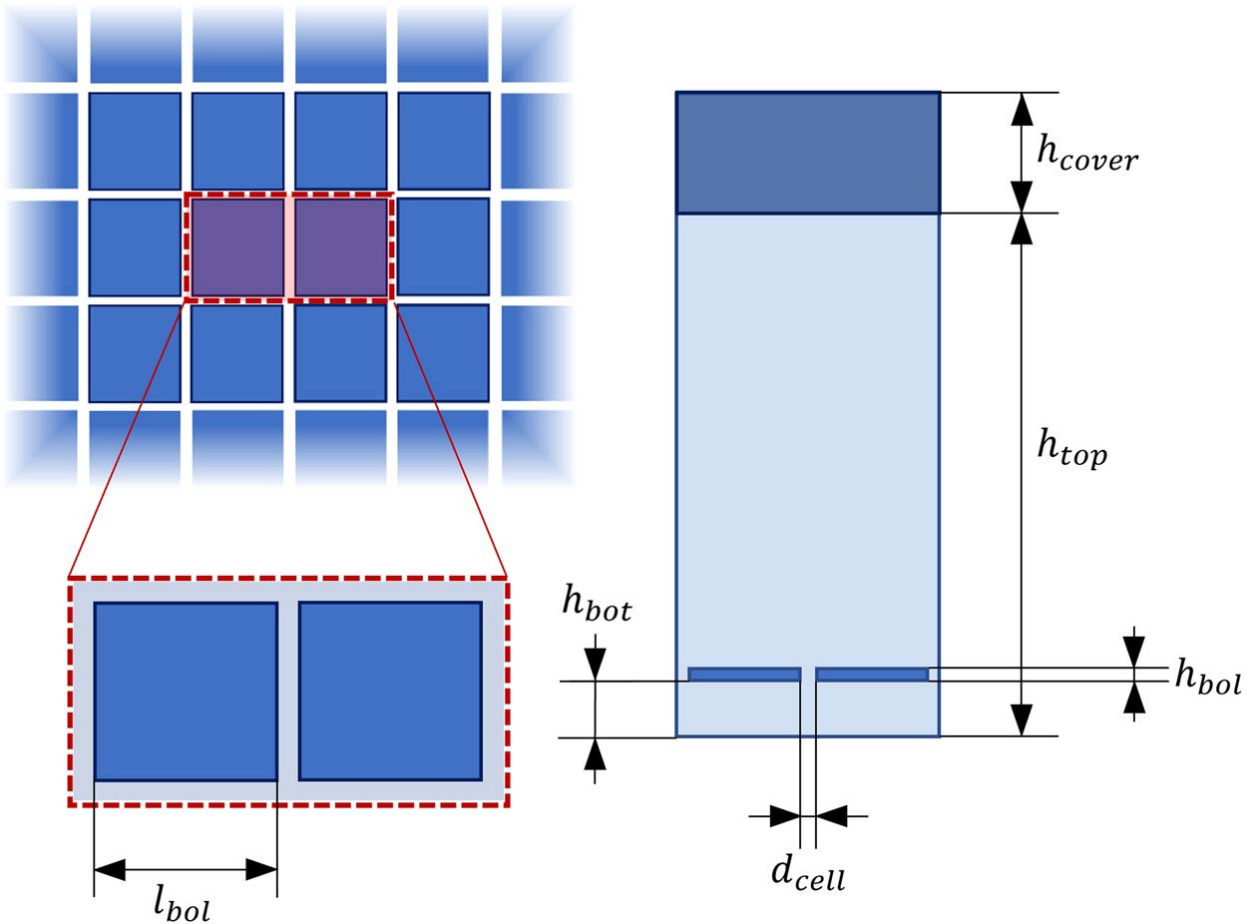
Sketches of the top and front views of the simulated unit cell. The values of the geometric parameters are: *l*_*bol*_ = 25 *μ*m, *h*_*bol*_ = 1 *μ*m, *d*_*cell*_ = 1 *μ*m, *h*_*top*_ = 97 *μ*m, *h*_*bot*_ = 2 *μ*m and *h*_*cover*_ = 10 *μ*m.

An array of microbolometers usually consists in several hundreds of silicon membranes regularly placed in both the x- and y-direction. Such elevated number of components makes possible the use of the periodic boundary conditions (PBC) for the lateral boundaries of the simulated domain, which allows us to simulate just the unit cell of the system instead of the whole of it. The DSMC-PBC act as an absorber for each incoming particle having an incident velocity *c*, for then recreating such a particle in the opposite boundary by preserving its velocity. The bottom boundary of the simulated domain is held at a fixed temperature ${T}_{W,B}=303.15\,{\rm{K}}$ since that’s the operating temperature of the electronic components of the device, while on the top wall of the unit cell, where a silicon cover layer of 10 *μ*m has been considered, the temperature (initially set to 293.15 K) freely evolves according to the Fourier’s law. The coupling between the two physics is made by the heat flux evaluated at the top boundary *q*_*top*_: what comes from the DSMC analysis represents the source term in the Fourier’s law, which in turn gives the temperature of the boundary *T*_*top*_. To model a real case study where the intensity of the incoming IR-radiation is not spatially constant, the unit cell contains two microbolometers, whose temperatures are different. This allows for the investigation of the heat flux along the lateral boundaries of the membrane for different ${\rm{\Delta }}T={T}_{bol,i}-{T}_{bol,j}$. For the investigated case, a single membrane has the dimensions of 25 × 25 × 1 *μ*m and the gap with its neighbour is 1 *μ*m. The simulated unit cell is shown in Fig. 1. The micro-cavity is supposed to be filled with argon (${m}_{p}=66.3\cdot {10}^{-27}\,{\rm{Kg}}$, ${d}_{m}=340\,{\rm{pm}}$) at the pressure $p=1\,{\rm{Pa}}$. The DSMC study has been carried out by considering 250000 simulated particles, tracked for 5000 time steps (observed to be enough for obtaining a steady state solution).

For what concerns the approximate diffusive analysis, conducted by using (7) for the thermal conductivity of argon, two 1D models for the characteristic dimensions *L*_*top*_ and *L*_*bottom*_ and one 2D model for the characteristic dimension *L*_*side*_ have been implemented, and a *β* = 1.5 has been used. The necessity of having a 2D model for evaluating the heat flux at the lateral sides of the microbolometers derives from their subtlety: the thermal field spreading in the thin gap between two adjacent membranes is strongly affected by the ones spreading towards the top and the bottom of the unit cell, leading to spatially varying heat fluxes.

**Figure 2:**
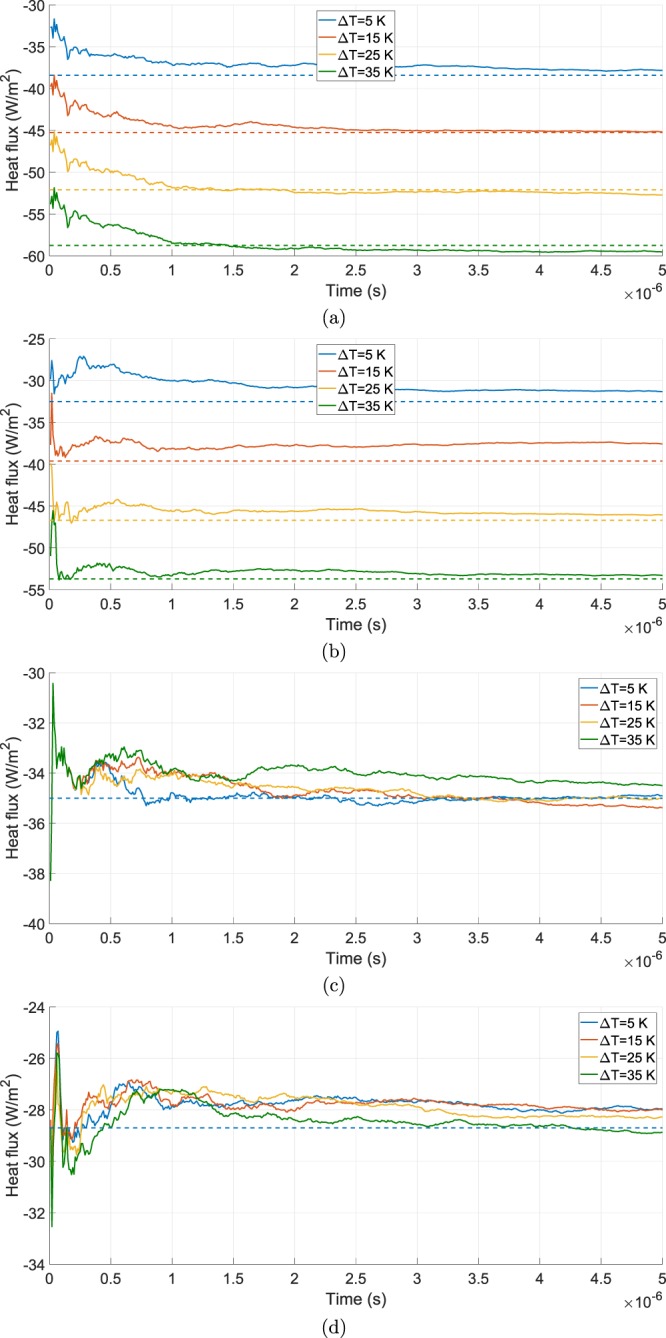
Comparison between the heat flux computed by the DSMC method (solid lines) and the one computed from the diffusive-approximate one (dashed lines) evaluated at the top (**a**) and bottom (**b**) surface of the hotter bolometer and at the top (**c**) and bottom (**d**) surface of the colder bolometer for different values of Δ*T*.

A first comparison between the heat fluxes obtained from the DSMC and the approximate diffusive approaches is shown in Fig. 2. Here, the parameter Δ*T* have been swept from 5 K up to 35 K, considering a fixed temperature for the second microbolometer of 343.15 K. The considerable difference in temperature between the membranes and the bottom surface of the micro-cavity *T*_*W*,*B*_ and between the two membranes Δ*T* is aimed at reducing the characteristic statistical noise of the DSMC approach. A good agreement between the solutions coming from the two different methods is observed both for the hotter and for the colder microbolometers, that is partially lost if to consider the heat fluxes obtained for the bottom surfaces for low values of Δ*T*: the committed relative error for the heat flux evaluated at the bottom surface of the hot membrane at Δ*T* = 15 K is 5.5%. A possible explanation for this comes from the statistical nature of the DSMC approach. Since the gap between the bottom surfaces of the microbolometers and the bottom surface of the micro-cavity is quite narrow, the number of incident particles used for the computation of the heat fluxes are probably not enough if to consider just 5000 time steps. Furthermore, the temperature difference between the membranes and the bottom surface of the micro-cavity is 10 K less than the one existing between the membranes and the top surface.

**Figure 3:**
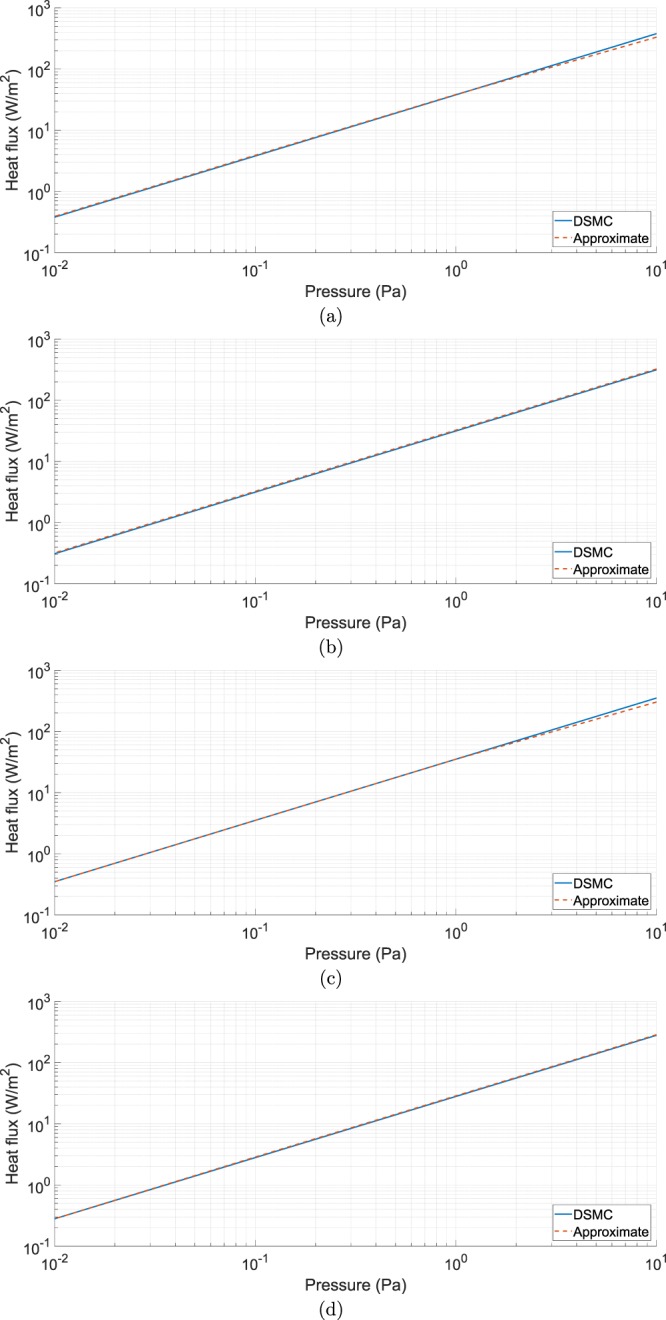
Comparison between the heat flux computed by the DSMC method (solid lines) and the one computed from the diffusive-approximate one (dashed lines) evaluated at the top (**a**) and bottom (**b**) surface of the hotter bolometer and at the top (**c**) and bottom (**d**) surface of the colder bolometer for different values of the pressure *p*.

Figure 3 shows the same comparison of Fig. 2 with the only difference that in this case Δ*T* is held constant at 5 K and the varying parameter is the pressure of the gas. The comparison between the obtained solutions is in general good also in this case, except for high pressure values, where the highest encountered relative error is 12.47%. The reason for this discrepancy is sought, also in this case, in the DSMC method: for a pressure of 10 Pa the Knudsen number falls to the collision case of the free molecular flow regime, which makes the implemented model insufficient.

**Figure 4:**
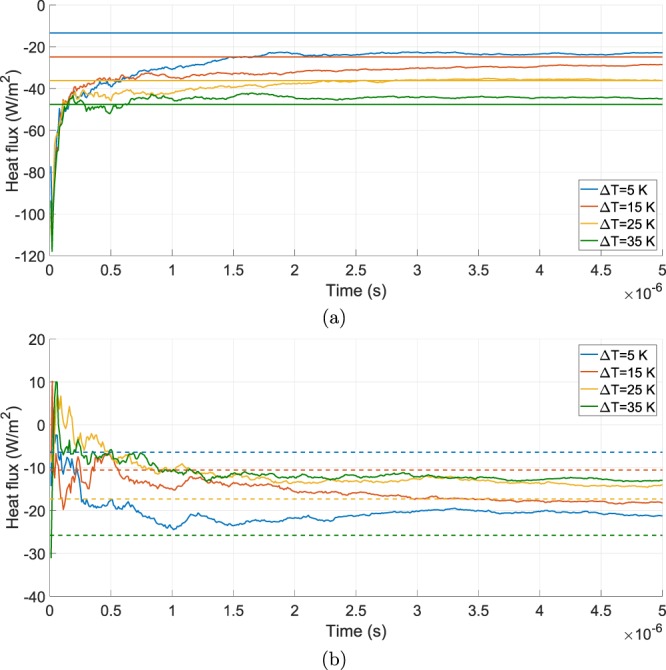
Comparison between the heat flux computed by the DSMC method (solid lines) and the one computed from the diffusive-approximate one (dashed lines) evaluated at the side walls of the hotter (**a**) and colder (**b**) microbolometers for different values of Δ*T*.

For what concerns the heat flux of the side walls of the microbolometers, Fig. 4 shows once again the comparison between the two approaches. As aforementioned, the diffusive-approximate model employed in the side walls case must be defined in a 2D environment, which gives spatially-varying heat fluxes for each value of Δ*T*. To give a constant value for the heat flux coming from the approximate approach, the spatial average has been taken. Apart from the high-Δ*T* cases of Fig. 4a, related to the hotter microbolometer, the approximate model fails in predicting the correct values for the heat flux. The most significant proof of this lies in the heat fluxes related to the colder bolometer, where the trend in respect to the variation of Δ*T* obtained from the approximate approach is the opposite of the one derived from the DSMC method. In this case the divergence between the results may be caused by the small value for the gap existing between two adjacent membranes and the strong variation of the thermal field. By observing the flat heat fluxe trends for the various simulated cases, we can deduce that the error introduced by the statistical nature of the DSMC approach seems to be quite negligible even though the number of particles hitting the lateral walls is definitely smaller in respect to the previous cases.

## Conclusion

By considering an array of microbolometers enclosed by a medium vacuum-level micro-cavity, a comparison between the data coming from the two methods of DSMC and Fourier’s equation for what concerns the investigation of the heat flux on the thermally active walls of the system has been presented. The DSMC approach, that represents the exact solution of the gas flow in the micro-cavity, makes use of the complete thermal accommodation theory by neglecting any kind of particle-particle interactions, while the approximate model is loaded with a modified expression for the thermal conductivity *k*, derived from the rarefied gas heat transfer theory. In general, a good agreement between the two approaches is obtained by varying the Δ*T* existing between the two membranes of the considered unit cell and the pressure of the micro-cavity. However, when it comes to consider the heat flux on the side walls of the microbolometers such agreement is mainly lost, with the approximate approach that fails in reproducing the results coming from the DSMC theory.
